# Three-dimensional evaluation of dental characteristics in patients with Cleidocranial dysplasia

**DOI:** 10.1186/s12903-024-04353-z

**Published:** 2024-05-17

**Authors:** Yang Lu, Jingfu Wang, Li Li, Xiaodong Zhang

**Affiliations:** Department of Stomatology, General Hospital of Northern Theater Command, No.83, Wenhua Road, Shenhe District, Shenyang, 110016 China

**Keywords:** Cleidocranial dysplasia, CBCT, Supernumerary teeth

## Abstract

**Background:**

Cleidocranial dysplasia (CCD) is an autosomal dominant hereditary disorder. Besides skeletal abnormalities, CCD is often associated with dental complications, such as multiple supernumerary teeth and permanent teeth impaction or delayed eruption.

**Methods:**

Supernumerary teeth of axial, sagittal and coronal CBCT view was characterized in detail and 3D image reconstruction was performed. Number and location of teeth, morphology of supernumerary teeth, positional relationship between supernumerary and adjacent permanent teeth, direction of supernumerary teeth in CCD patients were analyzed.

**Results:**

The mean age of the 3 CCD patients in this study was 16.7 years. Among 36 supernumerary teeth, the majority of them were identified as apical side located and lingual side located. Normal orientation was the most common type in this study, followed by sagittal orientation, and horizontal orientation. Horizontal orientation teeth were all distributed in the mandible. Supernumerary teeth exhibited significantly shorter crown and dental-root lengths, as well as smaller crown mesiodistal and buccolingual diameters (*P* < 0.01). There was no difference in the number of supernumerary teeth between the maxilla and mandible, and the premolars region had the largest number of supernumerary teeth and the incisor region had the smallest number.

**Conclusions:**

This study compares number and location of teeth, morphology of supernumerary teeth, positional relationship between supernumerary and adjacent permanent teeth and direction of supernumerary teeth, this study also provides a reference for the comprehensive evaluation of CCD patients before surgery.

## Introduction

Cleidocranial dysplasia (CCD) is an autosomal dominant hereditary disorder. CCD was named by Marie and Saintion In 1898 and the prevalence rate is about one in a million[1, 2]. The disease is caused by mutations in the runtrelated transcription factor (RunX2) gene on chromosome 6p21 Runx2 is transcription factors necessary for bone formation and responsible for controlling the differentiation of mesenchymal stem cells in osteoblasts, so mutations can lead to bone formation defects. CCD is characterized by skeletal abnormalities, including dysplasia of the clavicle (hypoplasia or hypoplasia), sutures and fontanelles, brachycephaly or a sunken bridge of the nose, hypoplastic maxilla and short stature. Besides skeletal abnormalities, CCD is often associated with dental complications, such as multiple supernumerary teeth and permanent teeth impaction or delayed eruption [3].

Although CCD is a form of generalized bone dysplasia, the extensive literatures are focused on dental problems because patients are more concerned about the appearance of their teeth. Studies have reported the number of supernumerary teeth ranging from 0 to 15 (average 7.5) [4] and 1 to 21 (average 8) [5]. In Jensen and Kreiborg‘s study, 18 of 19 patients had multiple supernumerary teeth, with frequencies ranging from 22% in the maxillary incisors to 5% in the molars. Supernumerary teeth were found in maxillary incisors (22.2%) and mandibular premolars (14.7%) [5]. Supernumerary teeth may also cause crowding, failure of eruption, diastema, root resorption, dilaceration and displacement of adjacent teeth [6].

Before the appearance of CBCT, intraoral, the panoramic radiographs, and surgically extracted teeth have always been the main data for the study of the supernumerary teeth [4]. It is logical to incorporate visualization of supernumerary teeth during the diagnosis and treatment planning phases. CBCT serves as a valuable resource, enabling the precise assessment of the morphology and positioning of supernumerary teeth, as well as their correlation with neighboring permanent teeth. Moreover, CBCT aids in determining the optimal surgical approach to minimize harm to the adjoining permanent tooth root and surrounding tissues [7]. However, few studies have evaluated the morphology and position of supernumerary teeth, especially through CBCT [8, 9]. Therefore, the objective of this study was to examine the shape and location of supernumerary teeth utilizing 3D imaging technique.

## Materials and methods

The following individuals, who were diagnosed with CCD and treated at the Hospital of Stomatology of China Medical University between January 2021 and December 2021, were selected for this study. Patients who underwent CBCT for diagnosis and orthodontic treatment planning were included, while those without supernumerary teeth or incomplete dental root development in their permanent teeth were excluded to eliminate any potential bias in evaluating the morphology and position of impacted supernumerary teeth in CCD. Only patients who had not undergone orthodontic treatment, tooth extraction, or fenestration of impacted permanent teeth, which could potentially affect the position, direction, and development of both supernumerary and permanent teeth, were selected. Prior to the CBCT procedure, patients were fully informed of its purpose and associated risks. All patients received treatment only after obtaining their own or guardian’s consent and signing the consent form. This study was approved by the Institutional ethics committee of School and Hospital of Stomatology, China Medical University (No. k20190052).

**Fig. 1:**
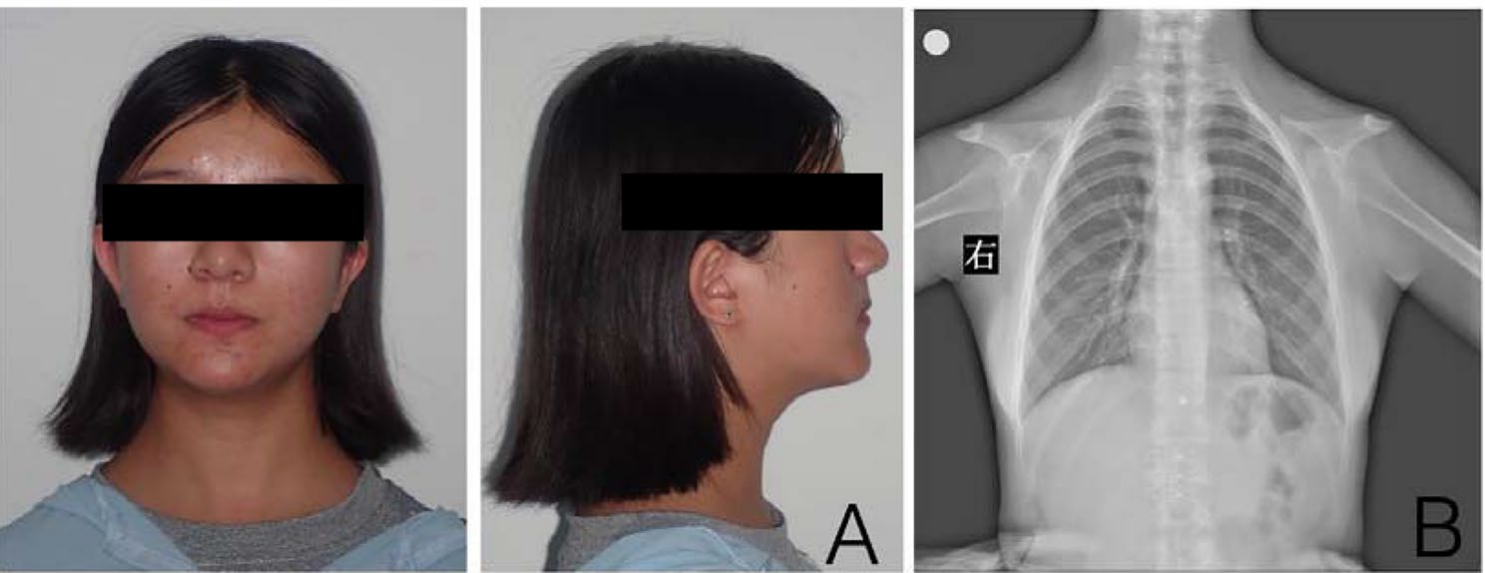
(**A**) Extraoral photographs showing the features of CCD patient: The skul is brachy-cephalic, with pronounced frontal and parietal bossings and the maxilla and zygomas are under developed. The bridge of the nose is broad and depressed, with ocular hyperterlorism; (**B**) Radiographic features of CCD subjects. Note the absence of clavicles shown by approximation of shoulders

**Fig. 2:**
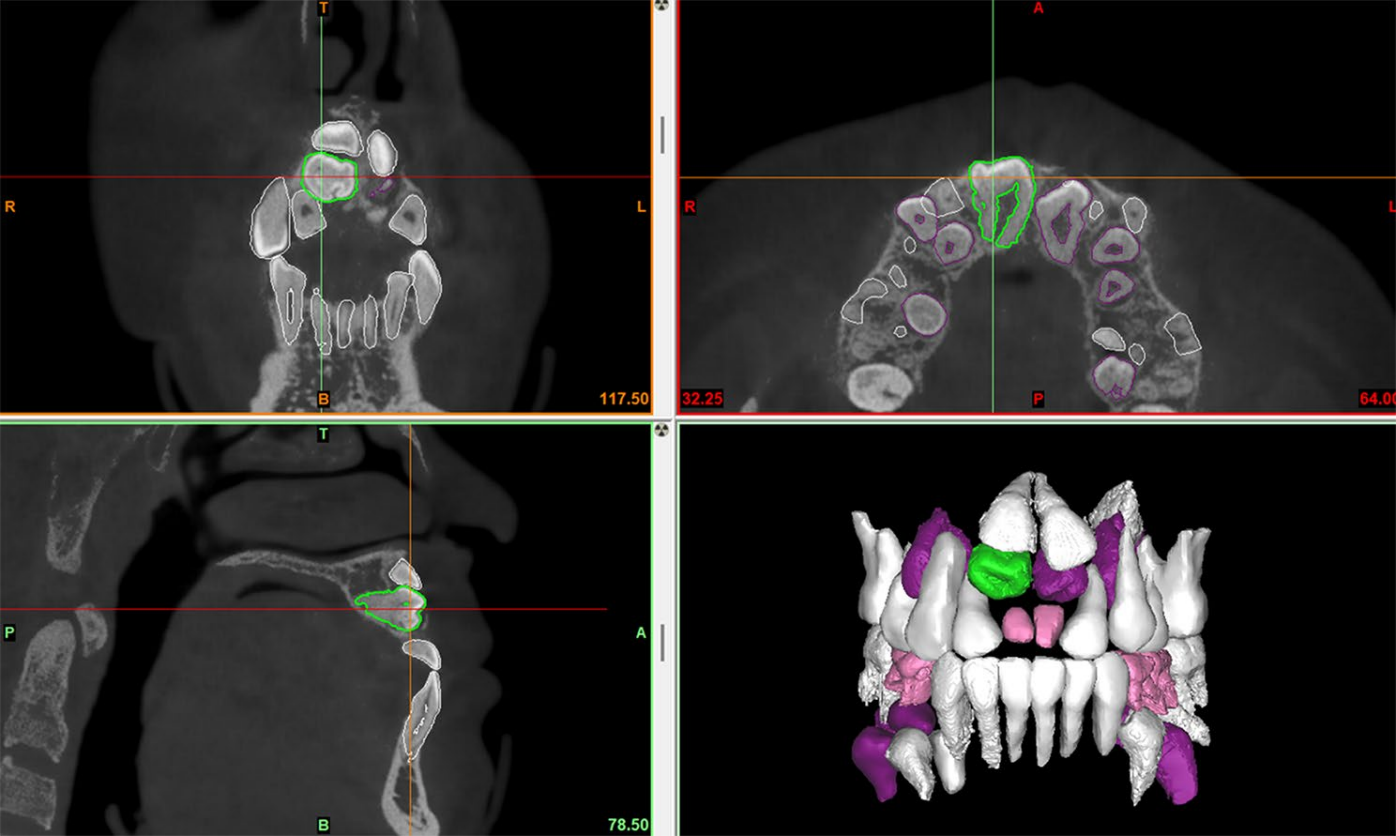
A coronal supernumerary tooth located in right anterior maxillary region (sagittal, coronal, axial sections of CBCT images and 3D image reconstruction)

**Fig. 3:**
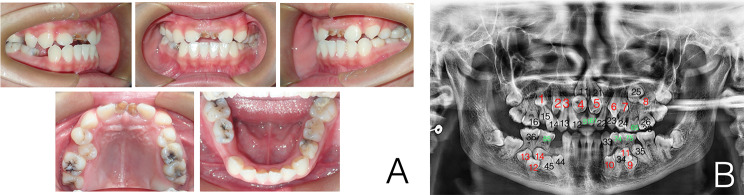
(**A**) Intraoral photographs showing the features of CCD subject, multiple retained primary teeth and multiple unerupted permanent and supernumerary teeth; (**B**) Panoramic tomography of CCD subject. Note the multiple retained deciduous teeth and their unerupted teeth (The red letter is for supernumerary teeth, the black letter is for permanent teeth, and the green letter is for primary teeth)

**Fig. 4:**
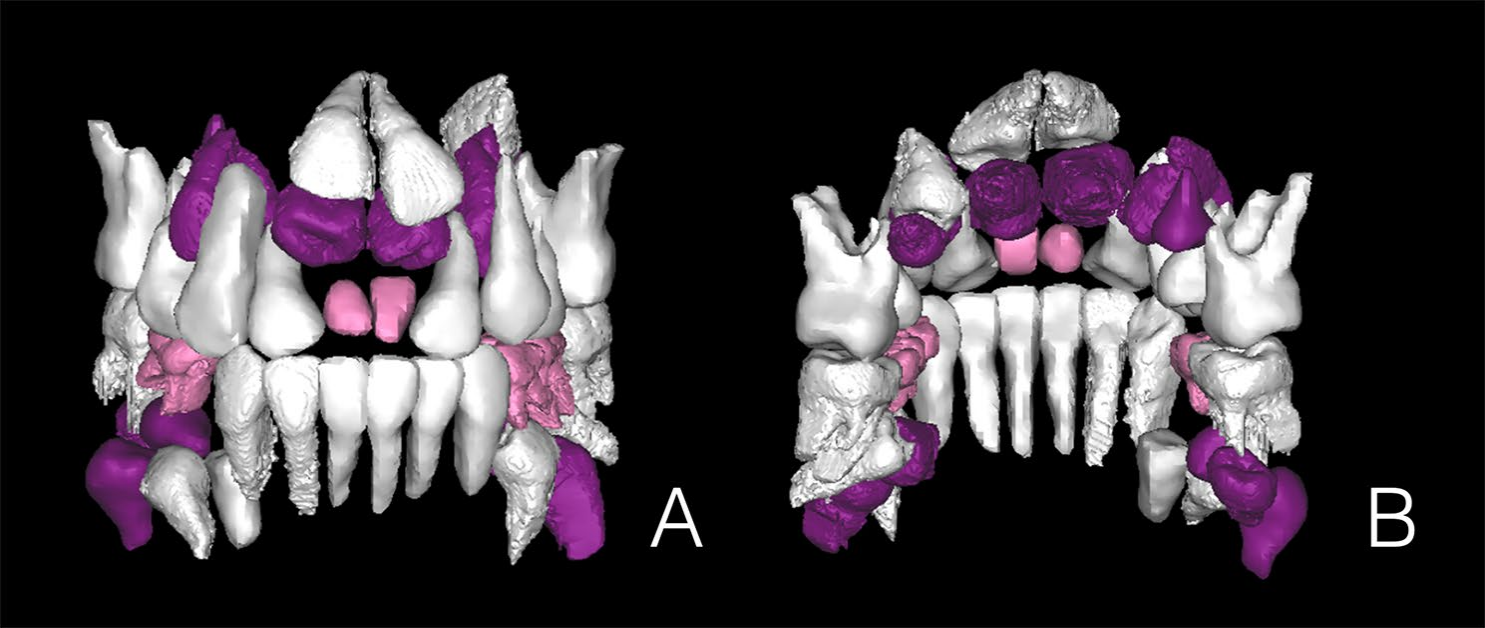
Three-dimensional images derived from cone-beam computed tomography data of teeth. (**A**) Labial and(**B**) lingual side views (Primary teeth, permanent teeth, and supernumerary teeth are shown in pink, white, and purple, respectively.)

Radiological investigation was performed with a same CBCT scanner (Sirona Dental Systems Inc., Bensheim, Hesse, Germany) by using the following settings: 6 cm x 16 cm field of view, 120 kV, 20.27 mA, 14.7 s exposure time and 0.25 mm slice thickness. Import the data into the Proplan 3.0 (Materialise, Inc., Belgium) software. Digital removal of maxillary and mandibular alveolar bone around teeth, and separated teeth from the whole image by threshold segmentation according to the difference of X-ray transparency. When the threshold is set at a range of -1024 to -328 Hounsfield units, the teeth can be effectively distinguished from other tissues. ST of axial, sagittal and coronal CBCT view was characterized in detail and 3D image reconstruction was performed (Fig. 2). Panoramic radiographs were traced, after which primary, erupted permanent, unerupted permanent, and supernumerary teeth were classified and color coded (Fig. 3). The primary teeth, eruptive permanent teeth, non-eruptive permanent teeth and supernumerary teeth were classified and color-coded by reference to panoramic X-ray films (Fig. 4). The CBCT images were initially assessed by a single examiner, a dentist who had received training in CBCT diagnosis.

Supernumerary teeth are defined as teeth or tooth-like structures that have erupted or remain impacted in the jaws, in addition to the standard 20 primary teeth or 32 permanent teeth. This condition is also known as one of the most common developmental anomalies in humans [10]. In this research, the identification of a supernumerary tooth is based on its proximity to the neighboring permanent tooth and differentiation from the permanent tooth is made through its position, inclination, crown shape, and root formation observed on panoramic radiographs. To avoid any confusion in distinguishing between impacted teeth (permanent teeth that have not erupted and supernumerary teeth) that are located closely to each other, the term “permanent teeth” is now assigned to impacted teeth with longer dental roots, while the term “supernumerary teeth” is assigned to impacted teeth with shorter dental roots. Furthermore, the term “adjacent permanent teeth” is used to refer to permanent teeth that are positioned nearest to their corresponding supernumerary teeth, including unerupted permanent teeth.

In all cases, the locations were divided into eight regions: anterior, canine, premolar, and molar regions in either the maxilla or mandible. The total number of teeth in each patient was recorded. Third molars were excluded from the tooth count in this study.

Conical shape was defined as a peg-shaped tooth with a normal root; Tuberculate was defined as a barrel-shaped tooth that has more than one cusp or tubercle; Supplemental shape was described as a tooth that resembles a normal tooth, and odontoma was defined as a mass of dental tissue that could not be described within the standard limits of tooth morphology [11, 12].

To determine the lengths of the dental crown and root, measurements were taken of the mesiodistal and buccolingual diameters of all supernumerary and adjacent permanent teeth. Following the methodology described in a previous study, crown length was defined as the distance from the midpoint of the cervical reference line (joining the labial and palatal cementoenamel junction) to the highest point of the crown, such as its cusp tip or incisal edge. Root length, on the other hand, was defined as the distance from the midpoint of the cervical reference line to the root apex [13]. The measurements were used to compare the groups of supernumerary and neighboring permanent teeth.

To compare the position relationship between supernumerary teeth crown and adjacent permanent teeth: coronal/apical, mesial/distal, labial/lingual.

The tooth axis was defined as a straight line connecting the central part of the occlusal surface, as described above, and the apex. The direction of supernumerary and unerupted permanent teeth was analyzed by the inclination angles of the tooth axis in relation to the occlusal plane. Normal was defined as the tooth axis being between 0 and 45 degrees from the longitudinal axis of the body in both coronal and sagittal sections. Inverted was defined as being upside down. Horizontal was defined as the tooth axis being between 45 and 90 degrees from the dental midline in the coronal section. Sagittal was defined as the tooth axis being between 45 and 90 degrees from the longitudinal axis of the body in the sagittal section. Others was defined as cases where the direction cannot be identified due to irregular tooth shape or resorption [14].

To minimize errors, the measurements were conducted on a weekly basis, repeated three times, and the average of the three measurements was obtained. The collected data was imported into SPSS Statistics, version 24.0 (IBM Corp., Armonk, NY, USA), for further analysis. Statistical significance was deemed significant at a p-value of less than 0.05.

## Results

The mean age of the 3 CCD patients in this study was 16.7 years, and their ages ranged from 16 to 18 years (male, 1; female, 2). Case 1 and Case 2 exhibited abnormal skull sutures. All patients had abnormal clavicles (Fig. 1 ), and their body height values were more than one standard deviation lower than age- and sex-matched Chinese norms reported previously. In terms of hereditary traits, Cases 1 and 3 had sporadic CCD, while Case 2 had familial CCD.

All subjects showed retained primary tooth, supernumerary, erupted permanent teeth, unerupted permanent teeth and supernumerary teeth (Table 1).

**Table 1:** Number and location of teeth of each case

		Incisor	Canine	Premolar	Molar	Total
Case 1	Retained primary tooth	2		3		5
	erupted permanent teeth	6	4	3	4	17
	unerupted permanent teeth	2		5		7
	supernumerary teeth	2	4	4	3	13
	Total	12	8	15	7	42
Case 2	Retained primary tooth		2	4	4	10
	erupted permanent teeth	6			4	10
	unerupted permanent teeth	2	4	4	2	12
	supernumerary teeth			4	2	6
	Total	8	6	12	12	38
Case 3	Retained primary tooth		1	5		6
	erupted permanent teeth				5	5
	unerupted permanent teeth	6	5	7	5	23
	supernumerary teeth	4	4	4	5	17
	Total	10	10	16	15	51
Total		30	24	43	34	131

The number of retained primary tooth in the three patients was 5, 10 and 6, respectively, with an average of 7 teeth. The number of erupted permanent teeth in the three patients was 17, 10 and 5, respectively, with an average of 10.7 teeth. The number of unerupted permanent teeth in the three patients was 7, 12 and 23, respectively, with an average of 14 teeth. The number of supernumerary teeth in the three patients was 13,6 and 17, respectively, with an average of 12 teeth.

In this subject, there was no difference in the number of supernumerary teeth between the maxilla and mandible, and the premolars region had the largest number of supernumerary teeth and the incisor region had the smallest number. The distribution of supernumerary teeth according to their position in the jaw is presented in Table 2.

**Table 2:** Number and location of teeth

		Incisor	Canine	Premolar	Molar	Total
Total	Retained primary tooth	2	3	12	4	21
	erupted permanent teeth	12	4	3	13	32
	unerupted permanent teeth	10	9	16	7	42
	supernumerary teeth	6	8	12	10	36
	Total	30	24	43	34	131

Morphological classifications of supernumerary tooth include the following 4 types: conical, tuberculate, supplemental, and odontoma. Conical and supplement were the common morphology (13 teeth). The morphology of supernumerary teeth is presented in Table 3.

**Table 3:** Morphology of supernumerary teeth

		Conical	Tuberculate	Supplement	Odontoma	Total
Maxilla	Incisor	2		2	2	6
	Canine	2	2			4
	Premolar	1			1	2
	Molar			5	1	6
	Total	5	2	7	4	18
Mandible	Incisor					
	Canine			4		4
	Premolar	6		1	3	10
	Molar	2		1	1	4
	Total	8		6	4	18
Total		13	2	13	8	36

To comprehensively characterize the positions of supernumerary tooth in a 3dimentional manner, we analyzed these teeth from Positional relationship between supernumerary and adjacent permanent teeth. Localization of the crown can be classified into three types as coronal/apical, mesial/distal or labial/lingual. Among 36 supernumerary tooth, 17 were identified as apical side located, 15 were identified as lingual side located, 2 were identified as labial side located, 1 was identified as coronal side, and 1 distally located. The positional relationship between supernumerary and adjacent permanent teeth is presented in Table 4.

**Table 4:** Positional relationship between supernumerary and adjacent permanent teeth

		Vertical position		Mesiodistal position		Labiolingual position		
		Coronal side	Apical side	Mesial side	Distal side	Labial side	Lingual side	Total
Maxilla	Incisor						6	6
	Canine				1		3	4
	Premolar					2		2
	Molar	1					5	6
	Total	1			1	2	14	18
Mandible	Incisor							
	Canine		4					4
	Premolar		10					10
	Molar		3				1	4
	Total		17				1	18
Total		1	17		1	2	15	36

In terms of orientation of supernumerary tooth, 4 subtypes such as normal, inverted, horizontal, and sagittal orientation were radiographically defined according to CBCT scan. Normal orientation was the most common type (24 teeth) in this study, followed by sagittal orientation (7 teeth), and horizontal orientation (5 teeth). Horizontal orientation teeth were all distributed in the mandible. Detailed data regarding Direction of supernumerary teeth of supernumerary tooth were listed in Table 5.

**Table 5:** Direction of supernumerary teeth

		Normal	Inverted	Horizontal	Sagittal	Total
Maxilla	Incisor	4			2	
	Canine	4				
	Premolar	1			1	
	Molar	4			2	
	Total	13			5	18
Mandible	Incisor					
	Canine	3		1		4
	Premolar	5		3	2	10
	Molar	3		1		4
	Total	11		5	2	18
Total		24		5	7	36

The findings from the size comparison between supernumerary and adjacent permanent teeth in each region revealed that supernumerary teeth exhibited significantly shorter crown and dental-root lengths, as well as smaller crown mesiodistal and buccolingual diameters compared to adjacent permanent teeth (*P* < 0.01).

## Discussion

The typical symptoms of CCD include craniofacial abnormalities, absent or hypoplasia of clavicles, narrow thorax, enlarged pubic symphysis, and knock-knees short stature [15]. The dental abnormalities serve as key indicators of CCD, with typical characteristics including the persistence of baby teeth, the presence of numerous supernumerary teeth, and the failure of permanent teeth to emerge. The study revealed a prominent feature of having a high number of impacted and supernumerary teeth. Additionally, other notable abnormalities encompassed the underdevelopment of the upper jaw, upward and forward rotation of the lower jaw, as well as a tendency towards skeletal class III malocclusion [16–18]. And it is important to note that CCD does not affect the intelligence of individual [19].

Due to the complex craniofacial deformity observed in CCD patients, there currently are no established guidelines for oral treatment. Creating a dental treatment plan for a CCD patient requires taking into account their physical appearance, age at diagnosis, as well as their social and economic circumstances. While developing a thoughtful treatment plan may be challenging, the objective of dental treatment remains clear: to attain the best possible functional and aesthetic outcomes while addressing the patient’s individual needs.

Some researchers propose that the lack of resorption of the overlying alveolar bone and mechanical hindrance from impacted supernumerary teeth are the primary factors responsible for the non-eruption of teeth in individuals with CCD [20]. Early surgical management with sequential exposure of the permanent teeth is an advisable approach. Once these teeth are uncovered, they will exhibit a typical pattern of eruption [21]. As a result, current treatment protocols typically initiate early interventions to facilitate the natural eruption of teeth with roots. After the removal of deciduous and supernumerary teeth that may impede the eruption, one should anticipate the expected growth of teeth with roots [22].

In order to better understand this syndrome and treatment options, and to better manage CCD patients, we conducted this study. In this study, number and location of teeth, morphology of supernumerary teeth, positional relationship between supernumerary and adjacent permanent teeth, direction of supernumerary teeth in CCD patients were analyzed. On panoramic radiographs, supernumerary teeth are typically differentiated from neighboring permanent teeth based on their position, inclination, crown shape, and root formation [4].

Among the subjects with CCD involved in this research, supernumerary teeth were found to occur in pairs alongside permanent teeth. While supernumerary teeth typically exhibit a tendency toward microdontia [23–26], the examination of crown morphology and size in anterior, canine, and premolar teeth depicted in 3D images demonstrated that supernumerary teeth in patients with CCD closely resemble their adjacent permanent teeth.

In the present study, the frequency of supernumerary teeth was the highest in the mandibular premolar region and the results correspond with those of previous reports [27]. Supernumerary teeth typically occur between primary and permanent teeth, implying that their presence could potentially impact the eruption of permanent teeth.

Supernumerary teeth in the anterior, canine, and premolar regions showed distinct resemblances in crown morphology to their adjacent permanent teeth, indicating a potential correlation with their location. The current findings provide further support for a previous discovery which observed similar resemblances between supernumerary teeth and their corresponding adjacent permanent teeth [28].

The relationship analysis between supernumerary and adjacent permanent teeth yielded the following findings: maxillary supernumerary teeth are predominantly located on the lingual side, while mandibular supernumerary teeth are mostly situated on the root side. Additionally, the supernumerary tooth exhibited a similar shape to that of the neighboring permanent teeth. However, the accurate size of the supernumerary tooth is affected by factors such as caries, root absorption, and irregular shape. Nevertheless, it is evident that the supernumerary tooth is smaller than its adjacent permanent teeth.

A previous study showed abnormalities in tooth morphology in patients with CCD [27], which were believed to be caused by limited space and hindered eruption. Specifically, crowding in the alveolar bone could affect the formation of dental crowns and roots. Regarding the orientation of supernumerary teeth, our study observed that in the maxillary incisor region, they tended to be oriented normally, while in the mandibular canine region, they were mostly horizontally positioned. Further research is needed to determine whether the abnormal orientation of supernumerary teeth is present during tooth-germ formation or if it occurs later in the development process due to insufficient space for tooth formation.

This study has limitations due to a small sample size, which prevents generalization of our findings, and the failure to examine the position and direction of supernumerary teeth at an early stage. The analysis of tooth in young patients at an early stage could provide insights into the position, direction, and order of development of supernumerary teeth. Nevertheless, this study is significant as it scrutinized various imaging features in CCD patients and identified features that could aid in diagnosis. Early diagnosis is crucial for CCD management since dental and maxillofacial treatment approaches depend on the patient’s age. Early diagnosis ensures that physicians do not miss the most appropriate treatment window considering the patient’s development and growth trajectory.

## Conclusion

The presented case demonstrated several common characteristics observed in CCD patients. Currently, there are still no established guidelines for treating CCD due to its rarity. Dental treatment for CCD patients is a complex task. Practitioners should be familiar with the various therapeutic options and select the most appropriate one based on the patient’s age, preferences, and compliance with treatment. CCD patients require a collaborative approach with effective communication and cooperation from the patient. Timing of intervention is crucial, and multiple surgeries may be necessary. Therefore, a comprehensive evaluation of the number and location of supernumerary teeth prior to surgery and a well-thought-out treatment plan are essential to achieve optimal results.

## Data Availability

The datasets used and/or analysed during the current study available from the corresponding author on reasonable request.
